# Complete Genome Sequence of a Pseudomonas simiae Strain with Biocontrol Potential against *Aphanomyces* Root Rot

**DOI:** 10.1128/MRA.00222-21

**Published:** 2021-05-06

**Authors:** Ashebir T. Godebo, Keith D. MacKenzie, Fran L. Walley, James J. Germida, Christopher K. Yost

**Affiliations:** a Department of Soil Science, University of Saskatchewan, Saskatoon, Saskatchewan, Canada; b Department of Biology, University of Regina, Regina, Saskatchewan, Canada; c Institute for Microbial Systems and Society, University of Regina, Regina, Saskatchewan, Canada; d Roy Romanow Provincial Laboratory, Saskatchewan Health Authority, Regina, Saskatchewan, Canada; Loyola University Chicago

## Abstract

Aphanomyces euteiches is a soilborne plant pathogen. It causes severe root rot in leguminous crop species. We report the complete genome sequence of a biocontrol strain, Pseudomonas simiae K-Hf-L9. The strain inhibited Aphanomyces euteiches mycelia and zoospores and suppressed root rot in field peas grown under controlled growth chamber conditions.

## ANNOUNCEMENT

The genus Pseudomonas includes species that demonstrate a great deal of metabolic diversity and are found in many habitats (1). Strains of Pseudomonas simiae are reported to exhibit biocontrol activity against fungal and oomycete pathogens via siderophore-mediated competition for iron and induction of systemic resistance in the host plant (2, 3). Pseudomonas simiae strain K-Hf-L9 was isolated from the soil of a pea field crop in Saskatchewan, Canada (4).

Pseudomonas simiae strain K-Hf-L9 was inoculated from frozen stock (50% glycerol, in −80°C storage) on half-strength Trypticase soy agar plates (half-strength Difco Trypticase soy broth plus 1.5% agar) and grown for 24 h at 28°C. Genomic DNA was extracted using the FastDNA Spin kit (MP Biomedicals) according to the manufacturer’s instructions. Genomic DNA library preparation and whole-genome sequencing were performed at the University of Regina (Regina, Canada). Genomic DNA was sheared using a g-TUBE (Covaris, Woburn, MA, USA) and a centrifugation protocol yielding an average DNA fragment size of approximately 8,000 bases (Eppendorf 5424 centrifuge and two 30-s centrifugations at 2,000 × *g*). A library was prepared for Nanopore sequencing using the ligation sequencing kit (SQK-LSK109) together with the native barcoding expansion kit (EXP-NBD103; Oxford Nanopore Technologies). Sequencing was performed using a MinION Mk1B device and a FLO-MIN106D (R9.4.1) flow cell. The fast5 files generated by the run were base called and demultiplexed after sequencing using Guppy version 4.4.1 and the high-accuracy (HAC) model.

MinION DNA sequencing data were evaluated using NanoPlot version 1.27.0 (5). Sequencing of P. simiae strain K-Hf-L9 yielded a total of 39,604 reads. Filtlong version 0.2.0 (https://github.com/rrwick/Filtlong) was used to generate two sets of filtered reads. Filtered set 1 included 35,496 reads that were greater than 1,000 bp long, representing the top 95% of sequences in the data set. Filtered set 2 included all reads (39,159 reads) that were greater than 1,000 bp. Trycycler version 0.3.3 (https://github.com/rrwick/Trycycler) was used to semiautomate the generation of a circular consensus genome assembly produced by the assembler programs Flye version 2.8.1-b1676 (6), miniasm version 0.3-r179 (7) with minipolish version 0.1.2 (8), Raven version 1.2.2 (9), and Redbean version 2.2.5 (10). Filtered set 1 was used to generate the assemblies, while filtered set 2 was used in all postassembly steps and during the final polishing step, which was performed using Medaka version 1.0.3 (https://github.com/nanoporetech/medaka) and its r941_min_high_g360 model. Then, the assembly was evaluated using QUAST version 5.0.2 (11). minimap2 version 2.17 (7), SAMtools version 1.9 (12), and mosdepth version 0.3.1 (13) reported 59.87-fold average sequencing coverage of the final genome assembly. The P. simiae K-Hf-L9 genome was annotated using the National Center for Biotechnology Information (NCBI) Prokaryotic Genome Annotation Pipeline (PGAP) version 5.1 (14). The genome has a consensus length of 6,199,521 bp and a G+C content of 60.28% (Table 1). Average nucleotide identity (ANI) analysis using JSpeciesWS (15) was used to compare the K-Hf-L9 genome to other sequenced genomes of Pseudomonas strains. Strain K-Hf-L9 has an ANI value of 99.3% with respect to the P. simiae type strain CCUG 50988 (16).

**TABLE 1:** Summary of the Pseudomonas simiae K-Hf-L9 genome characteristics

Characteristic	Value(s)
No. of contigs	1
Genome size (bp)	6,199,521
G+C content (%)	60.28
Total no. of genes	5,679
Total no. of CDSs^*a*^	5,587
No. of CDSs with protein	5,425
No. of RNA genes	92
No. of complete rRNAs (5S, 16S, 23S)	7, 6, 6
No. of tRNAs	69
No. of noncoding RNAs	4
Total no. of pseudogenes	162

a CDSs, coding sequences.

### Data availability.

This whole-genome shotgun project has been deposited in GenBank under accession number CP066169.2, BioProject number PRJNA662093, and BioSample number SAMN16076439. The raw reads are published in the SRA database under the accession number PRJNA662093.
